# Exploring the Potential for Steel Slags Valorisation in an Industrial Symbiosis Perspective at Meso-scale Level

**DOI:** 10.1007/s12649-022-01940-5

**Published:** 2022-10-13

**Authors:** A. Piemonti, A. Conforti, L. Cominoli, A. Luciano, G. Plizzari, S. Sorlini

**Affiliations:** 1 Department of Civil, Environmental, Architectural Engineering, and Mathematics (DICATAM), University of Brescia, 25123 Brescia, Lombardy Italy; 2 Department for Sustainability, ENEA (Italian National Agency for New Technologies, Energy and Sustainable Economic Development), Resource Valorization lab, Via Anguillarese 301, 00133 Rome, Lazio Italy

**Keywords:** Steel slag, Industrial Symbiosis, Mass Flow Analysis, Big data, Recovery, Reuse

## Abstract

A greater reuse of steel slags would bring considerable benefits both from an environmental and economic point of view. The development of tools and strategies to monitor at different scales resources and waste flows would allow for better resource planning and a more sustainable management on territory. The aim of this study is to investigate and analyse the supply chain that deals with the management of steel slags at meso-level, in order to investigate the state of implementation of industrial symbiosis (IS), its potential and its improvement. A Mass Flow Analysis (MFA) has been implemented, through big data analysis coming from the integration of regional and provincial databases with a careful data processing from questionnaires. This integrated methodology has proved to be a valid tool to monitor the recovery and reuse, the implementation of industrial symbiosis and to plan improvement actions. This paper reports a representation of the current situation regarding the production, recovery and reuse of these materials in production processes for which they are suitable, with a view to their full exploitation, following the principles of circular economy and an analysis of the mutual exchange that occur among steelmaking plants and other business partners in a network of industrial companies. The results showed that most of the steel slags managed at meso-level (Province of Brescia, Italy) is still unfortunately destined for landfill with low percentage of them classified as by-product highlighting as the IS is not adequately applied. Of the slag destined for treatments and recovery processes, almost all of them are Electric Arc Furnace slag, which are mainly reused for hydraulically bound base layers and road sub-bases (about 85% of the total recovered) and as aggregates for the production of cement and bituminous mixes (about 15% of the total recovered). Results shows as further effort should be made in term of policies and strategies to incentivize IS and to increase the recovery.

**Graphical abstract:**

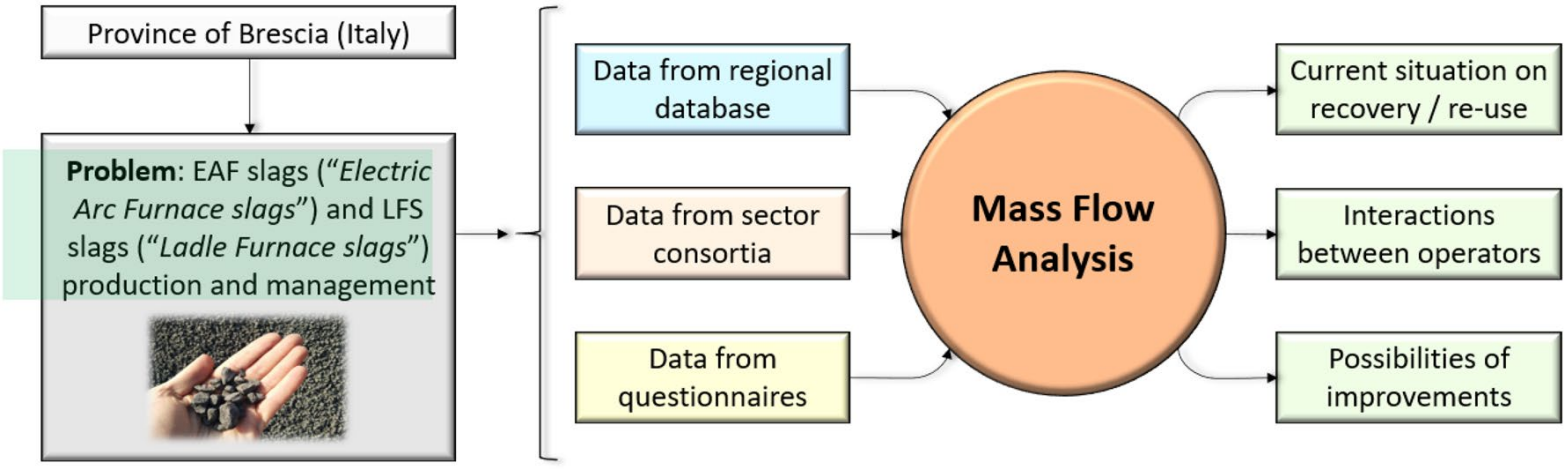

## Statement of Novelty

A tool for Mass Flow Analysis from big data has been developed and implemented at meso-scale to monitor and plan steel slags flows, their recovery and reuse, promoting industrial symbiosis.

## 1. Introduction

The global steel industry has seen a significant increase in production over the last 20 years, from 847.1 million tonnes in 2000 to 1,875.2 million tonnes in 2019. China has increasingly taken the lead in recent years, with a steel production accounting for around 50% of all steel produced internationally in 2019 [1]. Inevitably, 2020 was also a very complicated year for the steel sector, due to the SARS-CoV-2 pandemic. While China still managed to increase its production, albeit to a limited extent, many other leading steel producing countries (e.g. India, Japan, the US, etc.) experienced a sharp decline. These countries include Italy, with a decrease in total production of 12% compared to 2019 (from 23.2 million tonnes in 2019 to 20.4 million tonnes in 2020), continuing (and increasing) the negative trend that began in 2019 [1].

Steel production can mainly take place according to two distinct processes, integral and electric cycle, and, depending on the geographical area considered, one method may be predominant over the other. Italy can be considered an example. Between the end of the 20th and the beginning of the twenty-first century, Europe experienced a progressive transition from integral cycle (Blast Furnace BF and Converter BOF) to electric technology (Electric Arc Furnace EAF). Italy, compared to other countries, anticipated this transition. At that time, the Italian plants operating with integral cycle were all publicly owned, favouring size and high employment requirements over the profitability of the plant itself. On the other hand, the steel mills operating with electric cycle were all managed by private capital, which considered the electric option easier and more flexible. The decision to adopt EAF technology was also influenced by the scarcity of raw materials on Italian territory to supply the blast furnaces and the consequent necessity to invest in infrastructure to guarantee their handling and retrieval [2]. Today, about 85% of Italian steel is produced using EAF technology [1].

The integral cycle is divided into two steps: the first step is the production of pig iron in the blast furnace from raw materials such as iron ore, coke, limestone and other minor additions. Within the furnace, different reactions take place, which result in the formation of the primary material (molten pig iron) and the so-called “*Blast Furnace Slag*” (or “*BFS*”). The BFS is then tapped from the blast furnace, in a range of approximately 250–300 kg per tonne of pig iron produced. Depending on the cooling process, the BFS can be divided in three main types [3, 4]:

- GBS (“*Granulated Blast furnace Slag*”), produced after a quick cooling process with water to produce vitrified granulates, which can be used, after appropriate grinding treatments, in addition to concrete to partially replace the cement binder [5–9] or added to clinker for the production of Portland cement [10];
- ABS (“*Air-cooled Blast furnace Slag*”), produced after a slow cooling process with air to produce crystalline material. This type of BFS slag will then be ground, sieved and graded to produce aggregates for road construction and concrete [11–13];
- PBS (“*Pelletized Blast furnace Slag*”), produced after a quick cooling process with air to produce glassy or crystalline pellets. It is then subjected to grinding treatments and, depending on the grain size, can be reused as a partial replacement of clinker for the production of Portland cement or as aggregates for concrete [3].

**Fig. 1:**
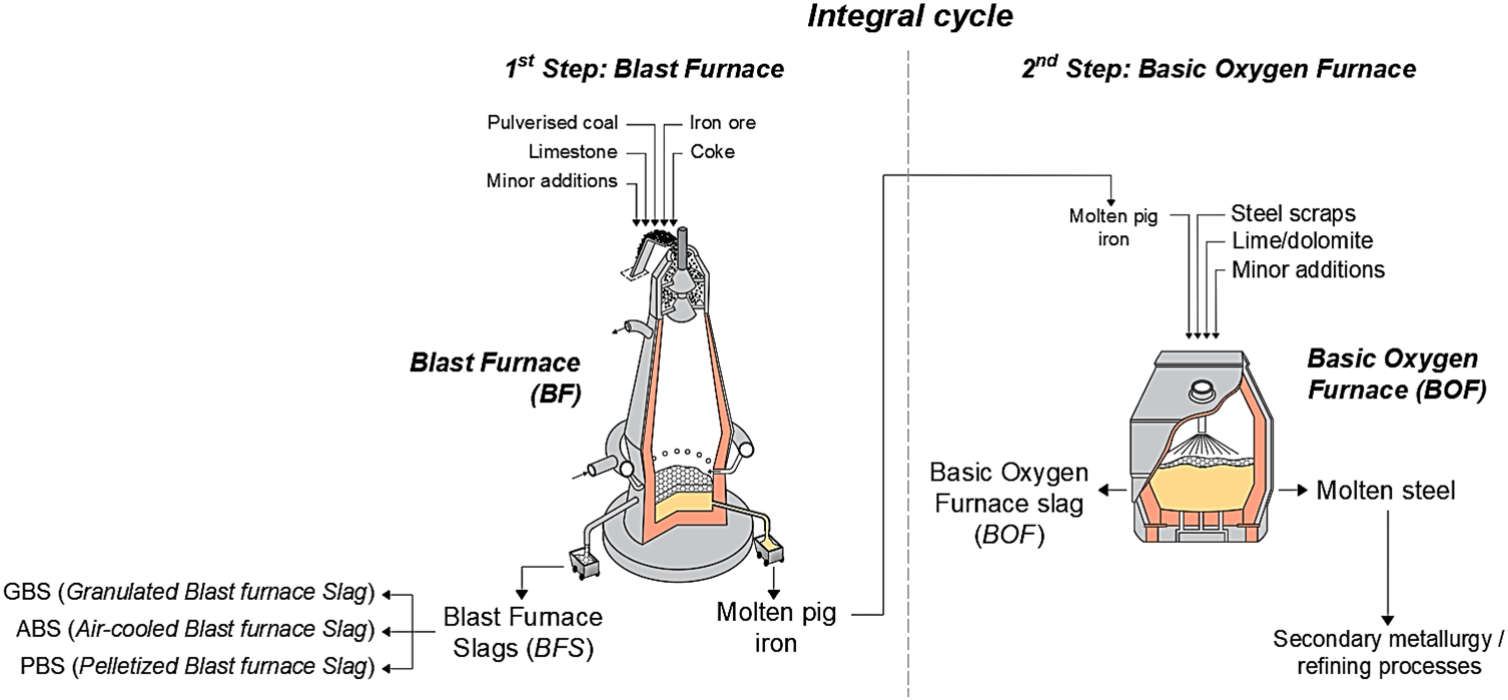
Summary of the two steps of the integral cycle. Image modified and adapted from [4]

The second step of the integral cycle consists in the production of steel in the oxygen converter from raw materials such as molten pig iron (from the blast furnace), steel scrap in small percentages (to maintain the temperature at 1600–1650 °C), lime/dolomite and other minor additions. In addition to the primary material (molten steel), the so-called “*Basic Oxygen Furnace slag*” (or “*BOF*”) derived from the combination of the impurities with lime and dolomite, is also tapped from the converter, in a range of approximately 100–150 kg per tonne of steel produced. This type of slag is mainly used as aggregate in road construction [3, 14–16], as well as other minor reuses in concrete production [17–19] or as an addition in fertilisers [20, 21]. Figure 1 shows, in summary, the two steps of the integral cycle.

**Fig. 2:**
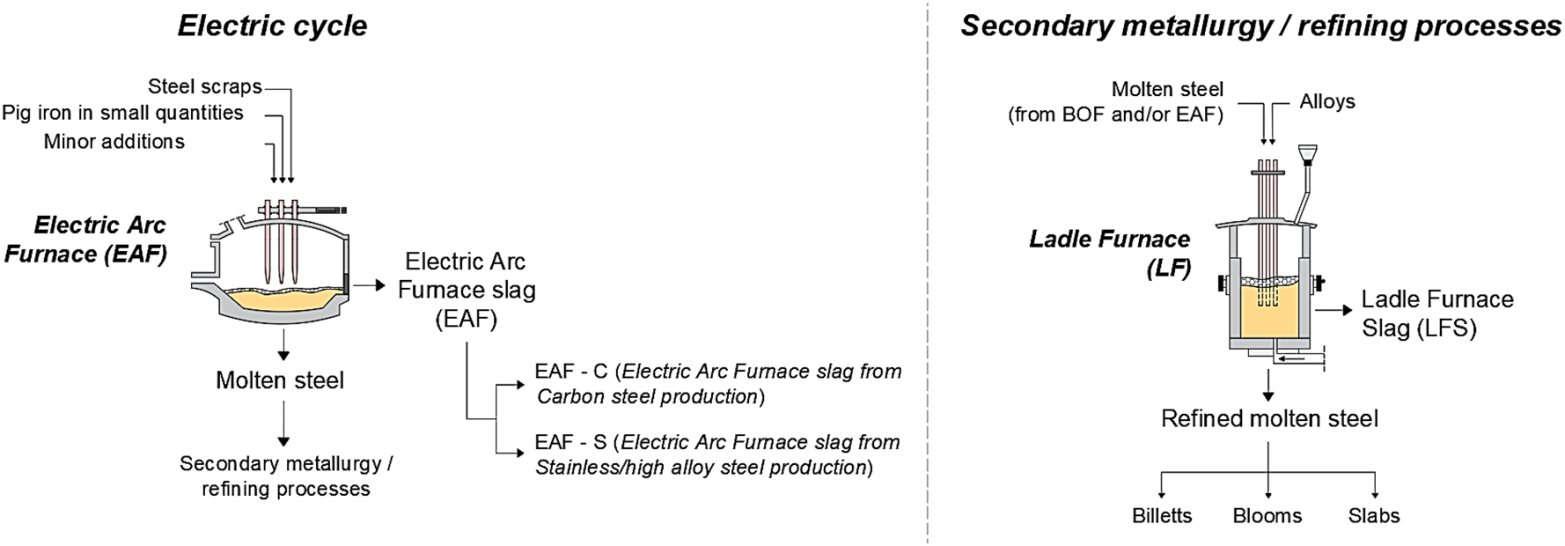
Summary of the two steps of the electric cycle and the secondary metallurgy process in the ladle furnace. Image modified and adapted from [4]

The electric cycle is the main alternative to the integral cycle for steel production: it consists of a single step in the electric arc furnace starting from raw materials such as steel scrap (appropriately divided and selected), pig iron in small percentages and other minor additions [4]. Within the furnace, different reactions take place, which result in the formation of the primary material (molten steel) and the so-called “*Electric Arc Furnace slag*” (or “*EAF*”), in a range of approximately 150–180 kg per tonne of steel produced. This type of slag is divided into two categories, depending on whether the steel produced is carbon steel (“*Electric Arc Furnace slag from Carbon steel*” or “*EAF-C*”) or stainless/high alloy steel (“*Electric Arc Furnace slag from Stainless/high alloy steel*” or “*EAF-S*”) and is mainly used as aggregate for road construction [16, 22–25] or as a partial replacement of cement [6, 7, 26, 27] and/or natural aggregate in concrete [28–42]. The steel produced in both processes is then usually destined for secondary metallurgy operations in ladle furnace (e.g. desulphurization, degassing, changes in composition etc.). These steel refining processes give rise to another type of slag, called “*Ladle Furnace Slag*” (or “*LFS*”), in a range of approximately 30–80 kg per tonne of refined steel. Because of its characteristics and heterogeneity, which make it very difficult to reuse, this type of slag needs to be studied in depth [6, 8, 24, 43–45]. Figure 2 shows, in summary, the electric cycle and the secondary metallurgy process in the ladle furnace.

## 2. General Overview of the Steelmaking Plants in the Lombardy Region and in the Province of Brescia

With a total steel production of about 23 million tonnes (2019, last useful data for comparison, before the SARS-CoV-2 pandemic threw the entire world economy into crisis), Italy was the second largest producer in Europe after Germany not considering Turkey (which is not classified in the “EU28” countries in the World Steel Association statistics [1]). Of these 23 million tonnes, approximately 19 million tonnes (80%) came from electric arc furnace production, making Italy the largest producer of electric arc furnace steel in Europe (not considering Turkey, as mentioned before). Considering a production range of 150–180 kg of electric arc furnace slag (EAF) per tonne of steel produced, slag amounted to around 3.25 million tonnes in 2019 (to which must be added around 1 million tonnes of slag from secondary metallurgy processes, assuming that all the steel undergoes refining).

Almost all electric arc furnaces and ladle furnaces are located in Northern Italy and in particular in the Lombardy region (where there are 19 steel mills). One of the most virtuous provinces concerning the Italian steel sector is undoubtedly the Province of Brescia (in Lombardy), which has 11 steel mills located on its territory, contributing approximately to the 25–30% of the national steel produced and refined. In parallel with the steel production, there is also the production of a large quantity of slags (EAF and LFS), with values around 1.1 million tonnes in 2019. Unfortunately, a considerable part of these slags is not recovered and is still disposed in landfills, despite their good performance properties and the various fields of reuse that have been almost consolidated and studied, generating numerous problems in the management of the material.

**Fig. 3:**
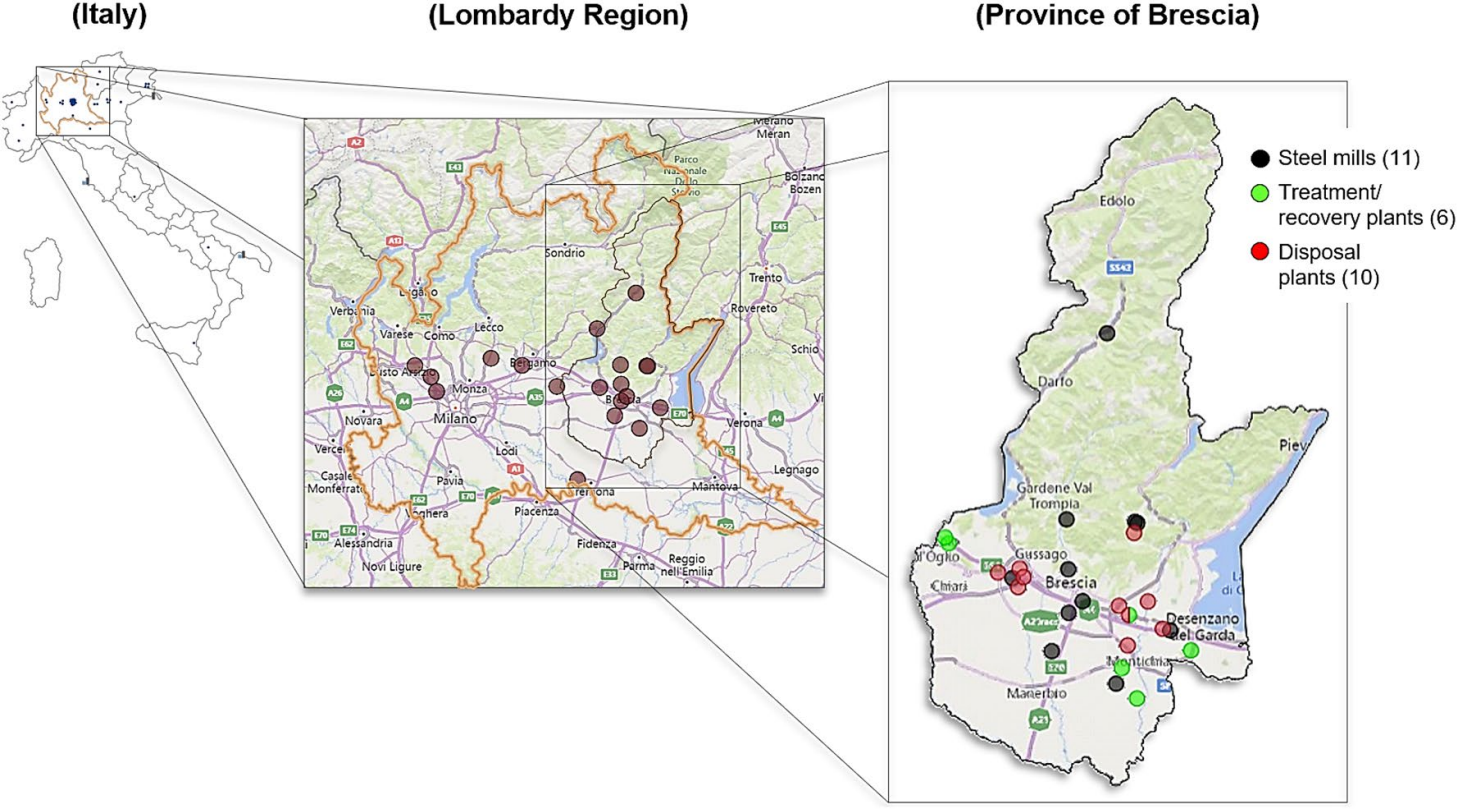
Location of the steel producers in Italy in 2019, with a focus on the Lombardy region and on the Province of Brescia. For the Province of Brescia, there is also a subdivision of plants into steel mills, treatment and recovery plants and landfills. Image modified and adapted from [46]

Figure 3 shows the location of the steel producers in Italy in 2019, including non-operational sites, with a focus on the Lombardy region and on the Province of Brescia (for the latter, there is also a subdivision of plants into steel mills, treatment and recovery plants and landfills) [46].

Their use as alternative materials for different application [5–45, 47] would be boosted, establishing regional Industrial Symbiosis (IS) agreements which can support companies to gain competitiveness in order to: reduce the environmental impact associated to their day to day business activities [48], utilizing platforms and systems to facilitate the identification of certified resources on the territory and their use also in the context of green public procurement [47, 49], utilizing tools developed for minimizing transport in resource and waste management [50].

The aim of this work is therefore to investigate and analyse the supply chain that deals with the management of steel slags in the Province of Brescia, from the producer to the final user and/or disposer, identifying the mass flows of these materials within the province, as well as the incoming and outgoing flows. This research aims not only to study the slags flows, but also to obtain a representation of the current situation regarding the reuse and reutilization of these materials in production processes for which they are suitable, with a view to their full exploitation, following the principles of the circular economy (increasing recovery, recycling and reuse of materials that would otherwise be destined for disposal, reducing land consumption, areas used for landfills, atmospheric emissions, etc.) and enhancing the paths of Industrial Symbiosis.

Symbiotic activities can be applied at different levels [51]: they can involve a single firm or organization (micro level), companies located in the same area (meso level) and finally the entire regional or national production system (macro level). Several Industrial Symbiosis applications have been developed at different scales [52] and different methodologies and tools have been developed and improved through the study of real applications to support IS implementation [53]. The greatest benefits are found to be achieved at meso level, where the clustering of complementary companies provides a complexity of functions [51, 54]. This is a situation compatible with the provincial dimensions and particularly with the productive system of the Province of Brescia, consisting of a broad complementarity and number of companies.

## 3. Data Analysis – Methodology

The Mass Flow Analysis is certainly a valid tool to understand not only how producers interface with transporters, recovery and/or disposal plants and final re-users, but also to identify the most appropriate applications to ensure greater recovery and to establish and promote Industrial Symbiosis at regional level. A Mass Flow Analysis has been implemented through big data analysis coming from the integration of available data on regional and provincial scales and through careful processing of data from questionnaires.

### 3.1. Regional and Local Scale Database Analysis

According to Italian legislation (Legislative Decree 152/2006 and subsequent amendments and updates [55]) and depending on the choices made by each steel mill, the slag produced can be classified either as “waste” or as “by-product”, with consequent differences in terms of management and treatment. The Lombardy region, through the ARPA (“*Agenzia Regionale per la Protezione dell’Ambiente*”), provides a database called MUD (“*Modello Unico di Dichiarazione ambientale*”), consisting of a set of declarations that all producers, transporters, treatment/recovery plants and disposers of waste must submit every year and in which waste is distinguished according to type, producer, origin and source [56]. For each of the 12 provinces of Lombardy, the database is divided into 29 tables, that can be exported in the desired format or consulted with special software, each containing one or more information and identified by two letters. Depending on the purpose of the research and the waste of interest, and using special filters, the database provides the desired information. The sections consulted for this research were:

- Section AA – “*Company and Local Unit Master Data*”, containing the master data of each company that has submitted the annual declaration of waste production, management, treatment or disposal. Among the most important data in this section, in addition to the name and the address of the company, there is the CIU (“*Codice di Identificazione Univoca provinciale*”): this is a code assigned to each declarant so that it can be easily and immediately identified in the other sections of the database;
- Section BA – “*Waste Communication*”, containing, for each reporting company, the main information on waste produced, managed, treated or disposed (total quantities produced and destined for third parties, stocks, etc.), subdivided by EWC code;
- Section BB – “*Attachments to Section BA*”, containing information on incoming and outgoing flows from each company. This section is subdivided into three different modules: module “*RT*” – “*waste received from third parties*”, module “*DR*” – “*waste delivered to third partied*”, module “*TE*” – “*waste transported by third parties*” and, for each module, the quantity of waste, the name and the address of the sender or receiver company are indicated;
- Section BD – “*Waste management, disposal operations*”, containing information on waste destined for disposal: quantities, name and address of the destination company and disposal category (in accordance with Annex B of Legislative Decree 152/2006 [55]);
- Section BE – “*Waste management, recovery operations*”, containing information on waste destined for recovery: quantities, name and address of the destination company and recovery category (in accordance with Annex C of Legislative Decree 152/2006 [55]).

The distinction between the different types of waste in the database is made possible by the assignment of an EWC code (from the European Waste Catalogue [57]), i.e. a sequence of three pairs of two digits, “*class*”, “*subclass*” and “*category*”, so that the waste can be identified according to production sector, production process, type and characteristics. As regards the steel slags examined in this study, they were classified by the producers using the following EWC codes:

- EWC code 10.02.01 – “*Waste from the processing of slag*”;
- EWC code 10.02.02 – “*Unprocessed slag*”;
- EWC code 10.09.03 – “*Furnace slag*”.

The MUD database is an appropriate tool for reconstructing the steps that a waste undergoes when it passes from the producer to the final user and/or disposer. However, this study was not only limited to the consultation of the data, but also aimed at simplifying their visualization: to this end, the data were first extrapolated and superfluous information were removed. This was followed by further processing, in order to represent and identify, in a quicker, simpler, more intuitive and interactive manner, all possible information contained in the database for the type of waste of interest.

### 3.2. Limitations of the Regional Database

Once tapped from the furnace and subjected to preliminary treatments (e.g. characterisation of composition, cooled according to different and proven methods, solidification, crushing, grinding, deferrization and division into piles [58]), the slags produced can be classified in two different ways, depending on company choices and availability:

- Slags classified with the status of “waste” (definition according to art. 183, paragraph a) of Legislative Decree 152/2006 [55]): they are subjected to the attribution of an EWC code by the producer, who is also required to fill in the MUD database annually, declaring quantities, production and destination. They will then be destined for recovery operations for a specific reuse (“End of Waste”, according to art. 184-ter of Legislative Decree 152/2006 [55]) or disposed in landfills;
- Slags classified with the status of “by-product” (definition and conditions according to art. 183, paragraph qq) and art. 184-bis of Legislative Decree 152/2006, respectively [55]). Compliance with the conditions of art. 184-bis, with the addition of the registration to the ECHA (“*European Chemicals Agency*”) and CE marking, allows the slags classified as “by-product” to be placed on the market and to be reused in sectors where they are most suitable, without undergoing “End of Waste” operations.

The analysis of the MUD database can provide a lot of information on the production and destination of steel slags in a given territory. However, by consulting the MUD alone, there is the risk of obtaining a representation that is not the exact representation of the real situation of the steel industry, due to some weaknesses in the database layout, that, unfortunately, contribute to providing useful but incomplete data:

- The database contains information only related to steel slags classified as “waste”; there is no information on slags classified as “by-products” (which, on further investigation, represent, on average, about 30–40% of the annual steel slags production);
- Although the database allows searches to be carried out using the EWC code of interest, it is not possible to distinguish between slag from electric arc furnace (EAF) and slag from ladle furnace (LFS). This is due to the fact that the two types of slag are classified under the same EWC code by most of the steel mills operating in the area, despite the fact that they differ greatly in terms of chemical, physical and mineralogical properties, performance characteristics and fields of application and reuse [4];
- By analysing sections BD and BE of the database, only partial information can be obtained on the disposal and recovery of slag classified as “waste”. In fact, disposal and recovery are only identifiable by the codes in Legislative Decree 152/2006, Annexes B and C of Part IV, from D1 to D15 for disposal and from R1 to R13 for recovery, respectively [55]. There is therefore no further information about the actual fields of reuse of the examined slags (e.g. road construction, concrete mixes, etc.).

### 3.3. Analysis of Big Data Obtained from Consortia and Operators in the Supply Chain

Given the above, further research was carried out in this study, trying to complete the weakness points in the database and provide a picture of steel slag management in the Province of Brescia that is as close to reality as possible. In order to do this, both the sector consortia and the operators in the Province of Brescia were directly involved, to obtain additional data to complete the weaknesses points in the MUD database outlined above. The first by means of discussion and in-depth meetings on the topic, the latter by sending questionnaires (customised for each company), with the aim of obtaining more information about the part of slag classified as a “by-product”, a subdivision of the slag produced between Electric Arc Furnace slag (EAF) and Ladle Furnace Slag (LFS) and an update of the data to the year 2020. In particular, steel mills and treatment/recovery plants were directly involved. While the participation of steel mills in the survey was not very active, remarkable results were obtained from the treatment/recovery plants (out of 6 recovery plants in the Province of Brescia, 4 actively participated in the data collection, representing about 80% of the total slags treated and recovered in the province). Consequently, it was possible to go into detail on the production and management of steel slag by subdividing the different quantities produced and treated in the Province of Brescia. For each year (2017 – 2020):

- Subdivision of the slags by classification: “by-product”, “waste” and “End of Waste”;
- Subdivision of the slags by type: Electric Arc Furnace slag (EAF or black slag) and Ladle Furnace Slag (LFS or white slag);
- Subdivision of the slags by origin: slag produced in the Province of Brescia, slag produced in the Lombardy region except for the Province of Brescia, slag produced outside the Lombardy region;
- Further subdivision of slag classified as “waste” according to recovery or disposal destination;
- Further subdivision of slags classified as “waste” and destined to recovery, according to EWC classification codes: 10.02.01 (waste from the processing of slags), 10.02.02 (unprocessed slags), 10.09.03 (furnace slags);
- Subdivision of slags according to the different reuse applications.

This made it possible to outline a situation much closer to the real situation regarding the production, management, treatment, recovery and disposal of steel slags in the province, compared to consulting only the MUD database.

## 4. Results

### 4.1. Results from the MUD Database Analysis

As mentioned in Paragraph 2 “General overview of the steelmaking plants in the Lombardy Region and in the Province of Brescia”, the Province of Brescia is located in the eastern part of the Lombardy region and is one of the most virtuous provinces in Italy as regards the steel sector. There are 11 steel mills on its territory, equipped with one or more electric arc furnaces and one or more ladle furnaces, depending on the company's management system. The total steel production in the Province of Brescia has undergone a significant increase in the last 10 years, reaching about 6 million tons in 2019. In parallel to this steel production, high quantities of slag are also generated, reaching about 825 thousand tonnes of electric arc furnace (EAF) slag and 285 thousand tonnes of ladle furnace (LFS) slag in 2019, for a total of about 1.1 million tonnes (the last useful year for a good understanding of the real quantities produced is 2019 because of the production crisis due to SARS-CoV-2 pandemic in 2020).

In the present paragraph, not only the overall production data, but also the slag flows in the province, from the producer to the final treatment/recovery plant and/or disposer, were analysed by consulting the MUD database for the last two years available: 2017 and 2018.

**Fig. 4:**
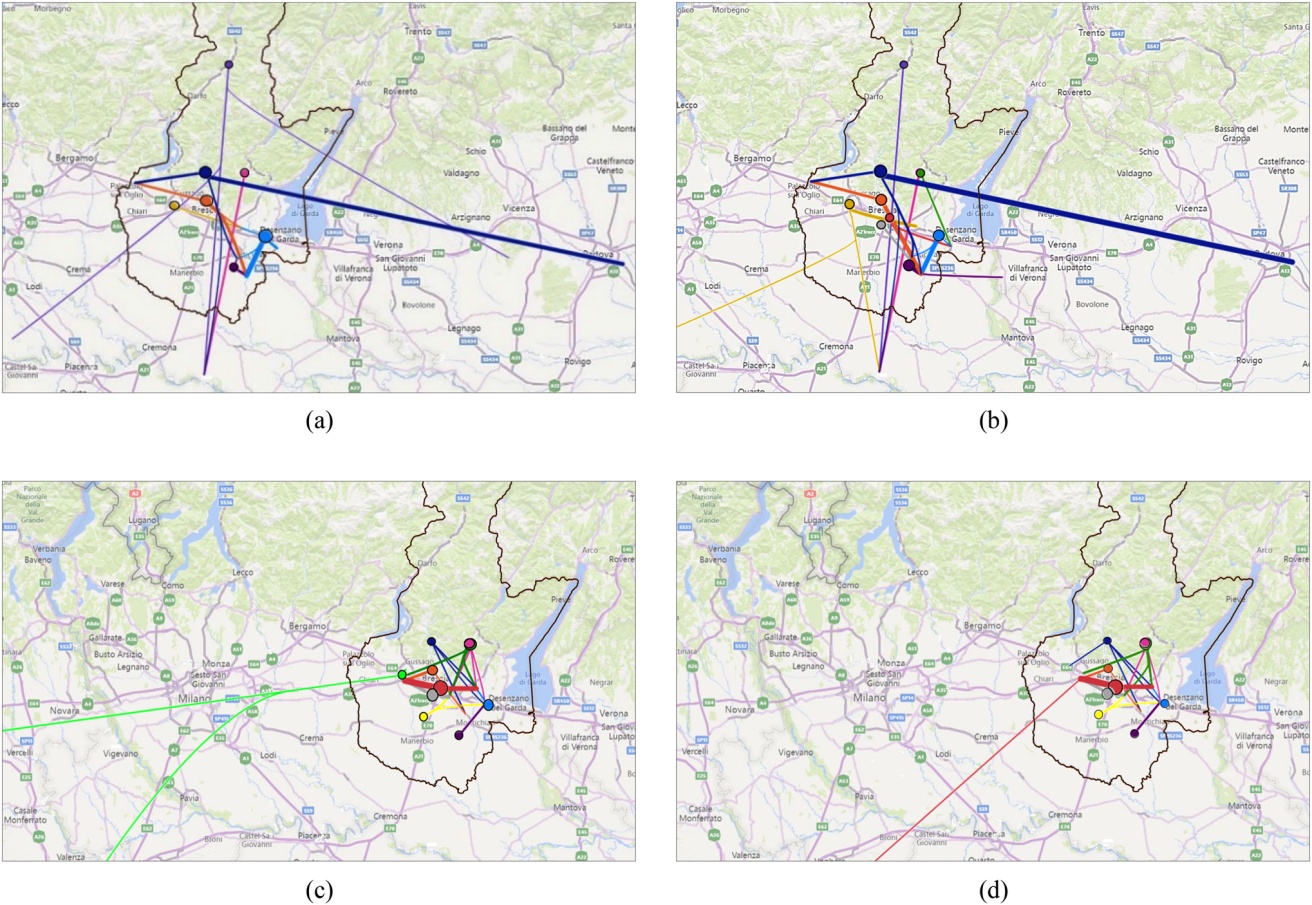
Example of processing showing the quantities of slag produced and destined for treatment operations, for the years 2017 (**a**) and 2018 (**b**), or for disposal in landfills, for the years 2017 (**c**) and 2018 (**d**). Treatment plants and landfills are located both inside and outside the province. Steel mills are marked with different coloured circles and a thicker line corresponds to a higher amount of slags going to a recovery plant

Further analysis and data processing made it possible to map the flows of steel slags produced in the Province of Brescia, highlighting both the quantities that remain within the province and those destined for recovery and/or disposal outside the province. Figure 4 shows an example of processing through which it was possible to identify the quantities of slag produced and destined for treatment operations at recovery plants or for disposal in landfills, located both inside and outside the province, for the years 2017 and 2018. In this figure, the steel mills are identified with different coloured circles for better distinction, and a thicker line corresponds to a larger quantity of slags going to a recovery and/or disposal plant.

Given the large amount of data analysed and processed, deriving from the consultation of the MUD database for the years 2017 and 2018, summaries of the quantities of steel slags produced both inside and outside the Province of Brescia and destined for recovery and/or disposal operations in the provincial territory, are explained below. In the following figures, the different quantities have been marked with letters for ease of visualisation:

- Letter “A”: production only of steel mills located outside the province;
- Letter “B”: production of other companies different from steel mills, located outside the province;
- Letter “C”: production only of steel mills located inside the province;
- Letter “D”: production of other companies different from steel mills, located inside the province.

**Fig. 5:**
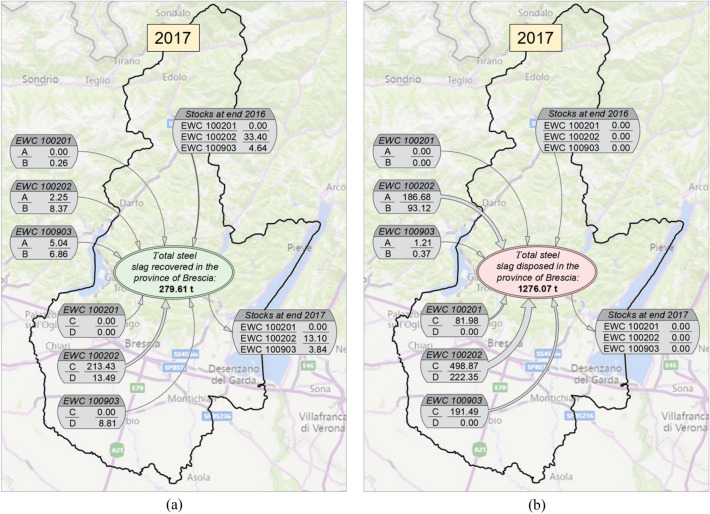
Summary of the quantities of steel slags produced both inside and outside the Province of Brescia (boxes located inside and outside the provincial boundary, respectively), subdivided by EWC classification code and destined for recovery (**a**) and disposal (**b**) in the provincial territory in 2017. **A** production only of steel mills located outside the province; **B** production resulting from other companies different from steel mills, located outside the province; **C** production only of steel mills located inside the province; **D** production resulting from other companies different from steel mills, located inside the province. Values in thousand tonnes (× 1000)

As shown in Fig. 5a, steel slags recovered in 2017 in the Province of Brescia amounted to 279.61 thousand tonnes. Approximately 80% (226.92 thousand tonnes) were slags with EWC classification code 10.02.02 from production in the Province of Brescia, which are divided into production from steel mills alone (a large part of the total, 213.43 thousand tonnes) and production from companies other than steel mills. The contributions of slags classified with the other two EWC codes analysed, from both production in and outside the province, made up a very small part of the total amount of recovered slags (approximately 7%). Figure 5b shows steel slags destined for landfill disposal in 2017 in the Province of Brescia. The total amounted to 1276.07 thousand tonnes. Unlike slags destined for recovery, in this case only 55% of the total were slags with EWC classification code 10.02.02 from the production in the provincial territory, which are divided into production from steel mills alone (498.87 thousand tonnes) and production from companies other than steel mills (222.35 thousand tonnes). In contrast to the case of recovered slags, significant contributions also derived from the production of slags classified as EWC 10.09.03 (191.49 thousand tonnes) and EWC 10.02.01 (81.98 thousand tonnes) by steel mills located within the province. As regards the slags produced outside the province, almost all of them consist of slags classified with EWC code 10.02.02, which made up about 20% of the total disposed.

From the analysis of the MUD database, for the year 2017, out of the total amount of steel slags classified as waste (EWC codes 10.02.01, 10.02.02, 10.09.03), managed in the Province of Brescia and deriving from production both inside and outside the province, unfortunately the quantity for disposal was significantly higher than that destined for recovery. The ratio “recovered slags to disposed slags” is approximately 0.18 (out of the total amount of slags classified as waste managed in the Province of Brescia, only 18% was destined for treatment and recovery, the remainder was destined for disposal in landfills).

**Fig. 6:**
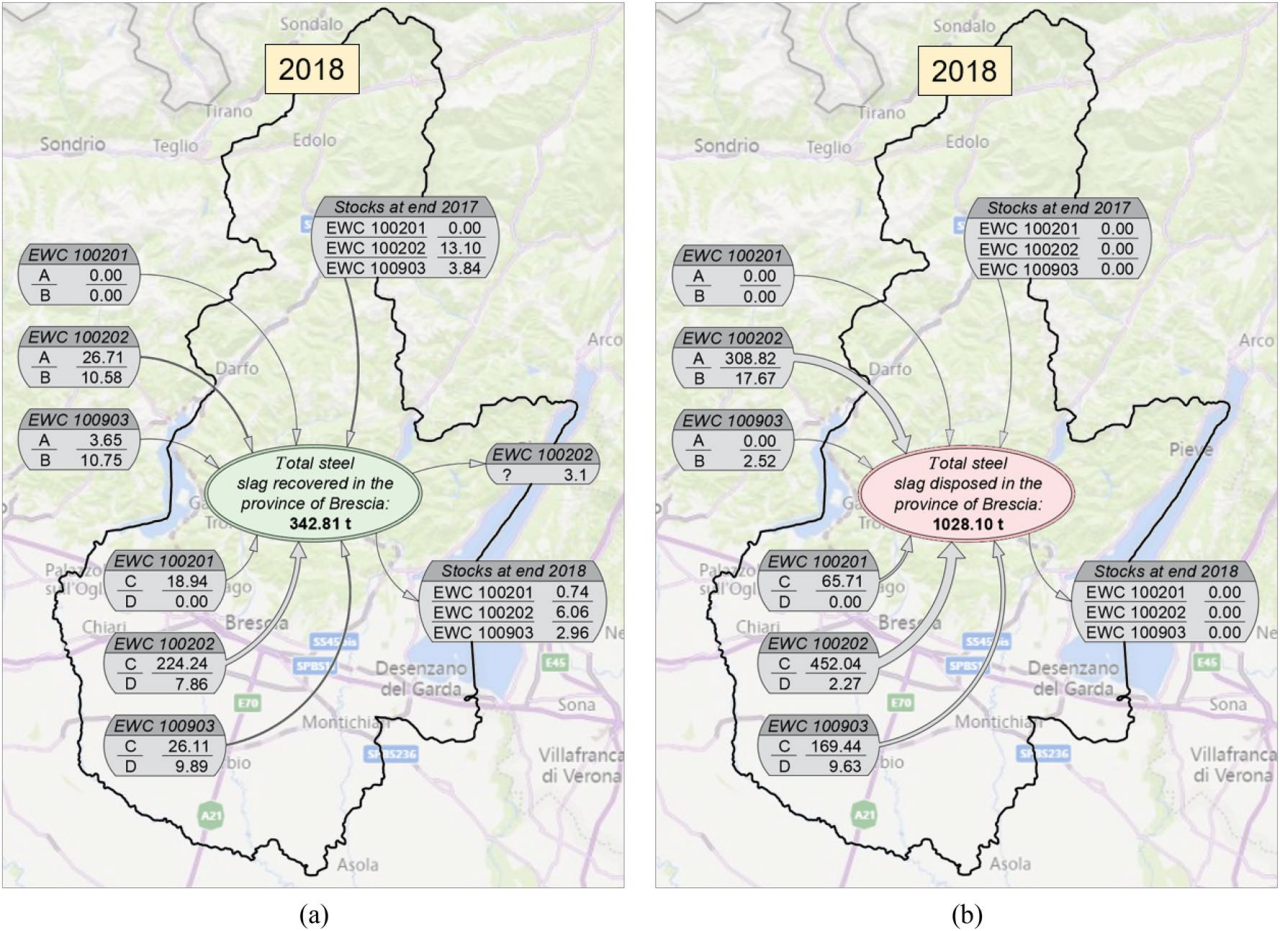
Summary of the quantities of steel slags produced both inside and outside the Province of Brescia (boxes located inside and outside the provincial boundary, respectively), subdivided by EWC classification code and destined for recovery (**a**) and disposal (**b**) in the provincial territory in 2018. **A** production only of steel mills located outside the province; **B** production resulting from other companies different from steel mills, located outside the province; **C** production only of steel mills located inside the province; **D** production resulting from other companies different from steel mills, located inside the province. Values in thousand tonnes (× 1000)

The same analysis was done for the year 2018, the latest available update of the MUD database.As shown in Fig. 6a, steel slags recovered in 2018 in the Province of Brescia amounted to 342.81 thousand tonnes (+ 23% compared to previous year). Approximately 68% (232.10 thousand tonnes) were slags with EWC classification code 10.02.02 from production in the Province of Brescia, which are divided into production from steel mills alone (a large part of the total, 224.24 thousand tonnes) and production from companies other than steel mills. The contributions of slags classified with the other two EWC codes analysed, from both production in and outside the province, made up the 20% of the total amount of recovered slags, higher than in 2017.

Figure 6b shows steel slags destined for landfill disposal in 2018 in the Province of Brescia. The total amounted to 1028.10 thousand tonnes (−19% compared to the previous year). Only the 44% of the total were slags with EWC classification code 10.02.02 from the production in the provincial territory, which are divided into production from steel mills alone (452.04 thousand tonnes) and production from companies other than steel mills (2.27 thousand tonnes). As in 2017, significant contributions also derived from the production of slags classified as EWC 10.09.03 (169.44 thousand tonnes) and EWC 10.02.01 (65.71 thousand tonnes) by steel mills located within the province. As regards the slags produced outside the province, almost all of them consist of slags classified with EWC code 10.02.02, which made up about 32% of the total disposed.

From the analysis of the MUD database, for the year 2018, out of the total amount of steel slags classified as waste (EWC codes 10.02.01, 10.02.02, 10.09.03), managed in the Province of Brescia and deriving from production both inside and outside the province, the quantity for disposal was still significantly higher than that destined for recovery. However, the ratio “recovered slags to disposed slags” has increased from 0.18 of 2017 to 0.25 of 2018.

It is clear that the amount of steel slags destined for disposal in landfills, compared to that destined to recovery operations, is unfortunately still very high. However, an analysis of the MUD database alone is not sufficient to provide a complete picture of the problem of the management of this material in the provincial territory, due to some limitations explained in the paragraph 3.2 “Limitations of the MUD database”.

### 4.2. Results from Sector Consortia Data: Impact of the Slags Classified as “by-Products”

From data obtained from a sector consortium (raMET, “*Consortium for environmental Research for Metallurgy*”), and declared by 10 out of 11 steel mills in the Province of Brescia, it was possible to complete, albeit not in great detail, the limitations of the MUD database described above. Several comparisons were made between the total quantities of Electric Arc Furnace slag (EAF) and Ladle Furnace Slag (LFS), as well as between slags classified as “waste” and “by-product” and between slags destined for recovery and slags destined for disposal. The time period taken into consideration was from 2017 to 2020. Table 1 shows the summary of the abovementioned data for EAF and LFS slags.

**Table 1:** EAF and LFS slag production in the Province of Brescia and subdivision into by-product, waste for recovery and waste for disposal (values in thousand tonnes). Reduced production in 2020 due to SARS-CoV-2 pandemic crisis

	EAF slag in the Province of Brescia				LFS slag in the Province of Brescia			
Year	Total production	By-product	Waste—recovery	Waste—disposal	Total production	By-product	Waste—recovery	Waste—disposal
2017	831.33	179.66	241.25	464.15	275.67	0.21	2.70	279.81
2018	824.96	92.02	317.22	415.73	280.57	0.00	3.15	277.40
2019	823.46	142.18	330.72	350.56	284.79	0.00	2.36	282.42
2020	674.60	121.85	369.17	183.58	229.14	0.00	1.24	233.90

**Fig. 7:**
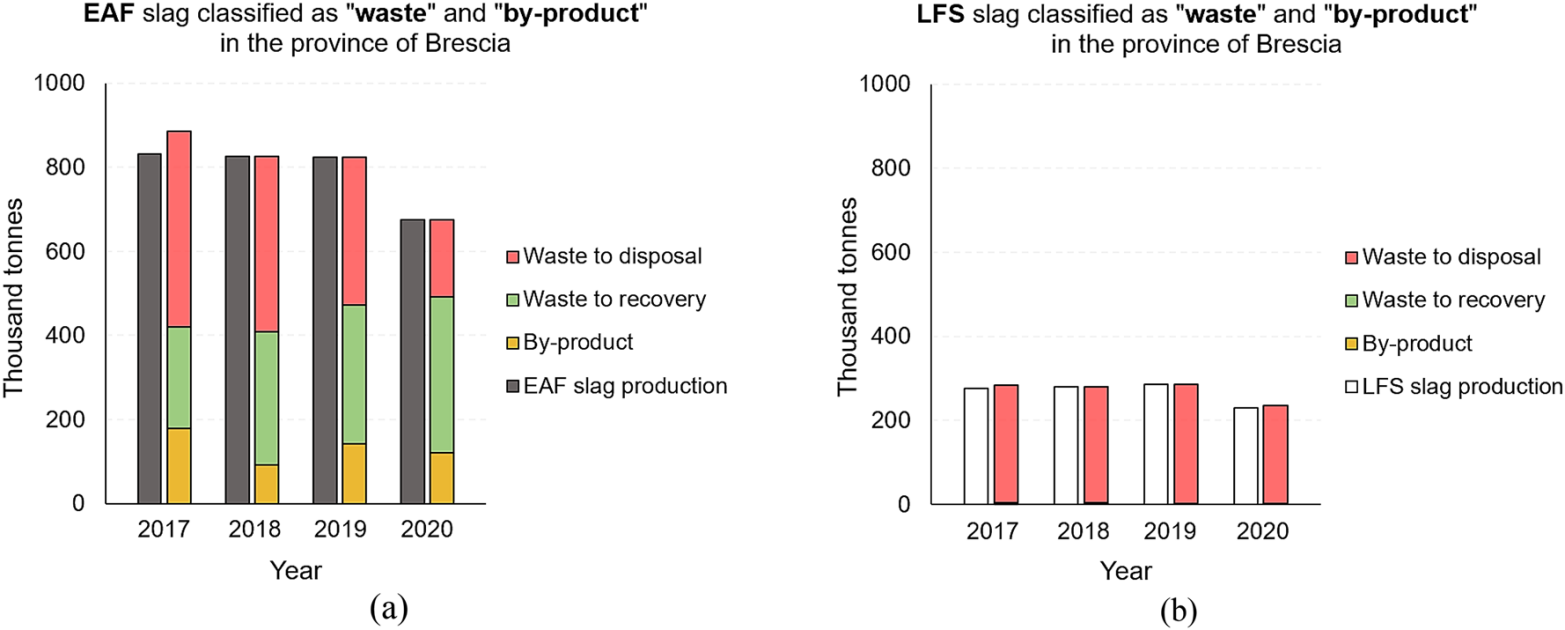
Comparison between the quantities of EAF slag (**a**) and LFS slag (**b**) classified as “by-product” and as “waste”, distinguishing between waste destined for recovery and for disposal, for the period 2017 – 2020 (production in 2020 is reduced due to SARS-CoV-2 pandemic crisis)

Out of the total slag produced in the Province of Brescia, about 80% is EAF slag (Fig. 7a) and the remaining 20% is LFS slag (Fig. 7b). This ratio remains almost constant for all years, due to the percentage of slag production per tonne of steel produced or refined [4]. Figure 7a shows a comparison between the amount of EAF slag classified with the status of “by-product” and that classified with the status of “waste”, expressed in thousand tonnes. For the latter category, a further subdivision was made according to whether the slag was destined for recovery or disposal. The same processing was carried out for LFS slag and the results are shown in Fig. 7b. Comparing the two graphs, a huge difference can be seen with regard to the classification, and consequently the management, of the two types of slags: EAF slag was classified as a “by-product” in percentages ranging from 10 to 20% of the total, depending on the year, while the percentages of LFS slag classified as a “by-product” were 0. With regard to the classification as “waste” destined for recovery, the percentages of EAF slag ranged from 30 to 55% of the total, depending on the year, while those of LFS slag never exceeded 1% of the total. Finally, a considerable difference can be noted with regard to the part of slag classified as “waste” and destined for disposal: while the percentages of EAF slag classified in this way have ranged from 27 to 52% of the total, depending on the year, those of LFS slag have always reached 99% of the total, denoting a reduced, if not null, demand for the material on the market and further highlighting the difficulties of the management, treatment and reuse of this type of slag. Even though EAF are recovered in higher percentage respect to LFS (ranging from 50-to 80% of the total production) and the recovery options are wide (aggregate for road construction [16, 22–25], partial replacement of cement [6, 7, 26, 27], natural aggregate in concrete [28–42]), the classification as by-product it still appear low highlighting critical issues still present in Italy that hinder producers in managing these residues as by-products. As consequence producers prefer divert their residues to dedicated treatment plants. The bottleneck is the difficulty of demonstrating the condition for by-products relating to the certainty of use (condition a) under the Waste Framework directive (WFD) article 5). In this context, industrial symbiosis initiatives at regional or national level, supported by a planning actions using MFA, will allow establish industrial symbiosis network among various companies in the road construction and rehabilitation sector [59] to achieve sustainable commercial opportunities for its members and to use resources efficiently.

### 4.3. Results from Questionnaires Data: Slags Managed by Treatment/Recovery Plants

**Fig. 8:**
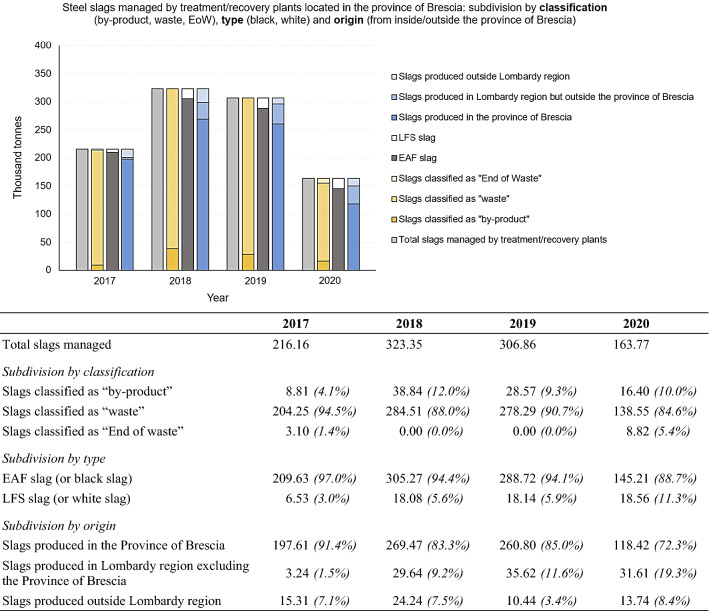
Steel slags managed by treatment/recovery plants located in the Province of Brescia: subdivision by classification (“by-product”, “waste”, “EoW”, type (EAF/LFS) and origin (produced in the province, produced in Lombardy region excluding the Province of Brescia and produced outside Lombardy region). Period 2017 – 2020. For a better comprehension, the figure is accompanied by a table showing numerical values (in thousand tonnes) and percentages of the total, for each subdivision made

Through the analysis of the MUD database and the processing of data obtained from the steel sector consortia in the Province of Brescia, a general picture of the production and management of steel slags in the province was obtained. The next step in this research was to involve companies directly by sending out data request questionnaires, in order to go into detail especially regarding treatments, recovery and reuse activities. In particular, steel mills and treatment/recovery plants were involved and the processed data obtained from questionnaires are shown below. Figure 8 shows the total amount of steel slags managed by treatment/recovery plants that participated in the survey, located in the Province of Brescia, for the period 2017 – 2020. For each year, the total amount of slags is divided according to classification (“by-product”, “waste”, “End of Waste”), type (EAF/LFS) and origin (produced in the province, produced in the Lombardy region excluding the Province of Brescia or produced outside the region). For a better understanding, the figure is accompanied by a table showing numerical values and percentages of the total, for each subdivision made. Values are in thousand tonnes. It is clear that most of the slag treated by treatment/recovery plants are classified as “waste” and comes from electric arc furnace steel production (EAF) within the Province of Brescia.

#### 4.3.1. Recovered Slags Classified as “Waste”

**Fig. 9:**
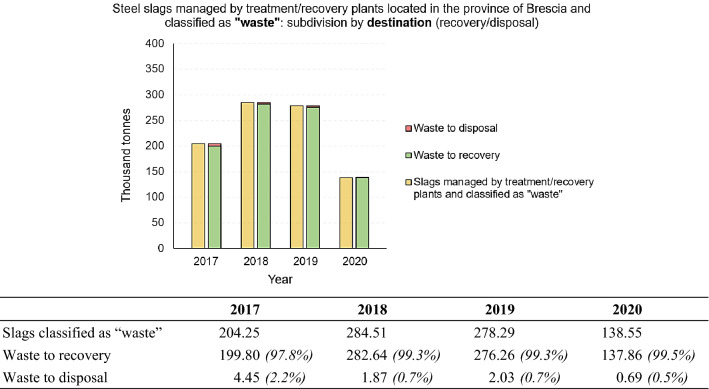
Steel slags managed by treatment/recovery plants located in the Province of Brescia and classified as “waste”: subdivision by destination (recovery or disposal). Period 2017 – 2020. For a better comprehension, the figure is accompanied by a table showing numerical values (in thousand tonnes) and percentages of the total, for each subdivision made

In the following, the subdivision by classification will be analysed in detail. Going into detail about the slags classified as “waste”, Fig. 9 shows their subdivision according to whether they are destined for recovery or disposal. Since these are treatment/recovery plants, the percentage of slags destined for disposal in landfills is obviously more than negligible.

**Fig. 10:**
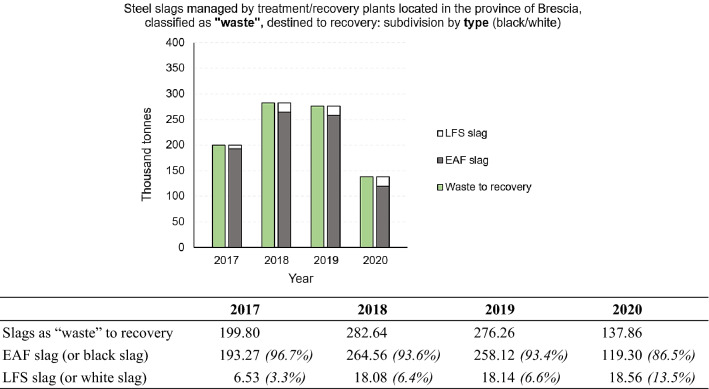
Steel slags managed by treatment/recovery plants located in the Province of Brescia, classified as “waste”, destined to recovery: subdivision by type (black slag or EAF/white slag or LFS). Period 2017 – 2020. For a better comprehension, the figure is accompanied by a table showing numerical values (in thousand tonnes) and percentages of the total, for each subdivision made

The slag classified as “waste” and destined for recovery was then further subdivided according to type, EAF and LFS (Fig. 10) and further processing were carried out for each type. Figure 10 confirms, once again, that slag from steel production in the electric arc furnace (EAF) represents the vast majority of the slags destined for recovery. Due to their good physical, chemical and mechanical properties, EAF slag can be compared to the natural aggregate and thus be suitable for various reuses in the construction sector [4, 58] among which the most widespread and consolidated are the cement replacement material and the replacement of fine or coarse aggregates in concrete and asphalt production. In addition to their use in the standard concrete application, few studies on the reuse of the EAF slag as a partial substitution of the natural aggregates in special concretes are available in the literature: pervious concrete, high-strength concrete [28], self-compacting concrete [39], and fiber reinforced concrete (using both steel and synthetic fibers) [40]. The recovery of LFS is less widespread, due to its dusty appearance [60, 61].

**Fig. 11:**
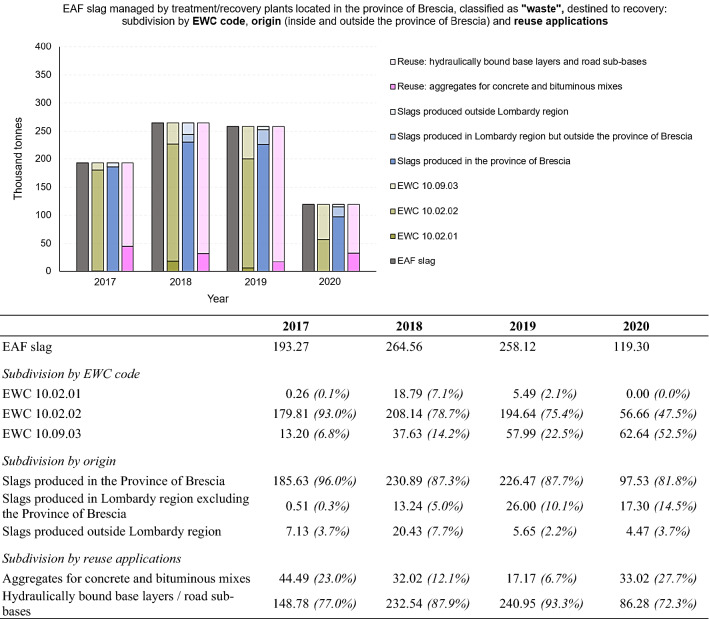
EAF slag managed by treatment/recovery plants located in the Province of Brescia, classified as “waste”, destined to recovery: subdivision by EWC code (10.02.01, 10.02.02, 10.09.03), origin (produced in the province, produced in Lombardy region excluding the Province of Brescia and produced outside Lombardy region) and reuse applications. Period 2017 – 2020. For a better comprehension, the figure is accompanied by a table showing numerical values (in thousand tonnes) and percentages of the total, for each subdivision made

Figure 11 shows the data processing carried out with regard to EAF slag (or black slag) destined for recovery. They were subdivided by EWC classification codes, origin and reuse fields. While the quantities classified with EWC code 10.02.021 (“*waste from the processing of slags*”) can be considered negligible, it can be seen that most of them are classified with EWC code 10.02.02 (“*unprocessed slags*”). The percentage of EAF slag classified with EWC code 10.09.03 (“*furnace slags*”) shows an increasing trend from 2017 to 2020. As expected, most of the EAF slag destined for recovery in the Province of Brescia comes from production within the province, with negligible quantities imported from outside the province and the region. As regards the possible applications, they undergo different treatment processes, in order to guarantee their suitability for different reuse fields [58]:

Road construction (unbound road embankments and sub-basements);

- Landfill capping;
- Hydraulically bound base layers;
- Aggregates for concrete production: low-performance concretes (with low cement dosages, for sub-foundations, foundations and basements), ordinary concretes, industrial concrete floors (where tensile/bending strength and durability are very important), precast concrete products, heavy concretes;
- Aggregates for bituminous mixtures and surface treatments for roads and traffic areas (e.g. airports);
- Innovative reuses being tested and studied (application in polymer matrices, 3D printing, EAF slag fillers in the field of plasma-applied hard covering, etc.).

Figure 11 shows that most of the EAF slag treated and recovered in the Province of Brescia are reused for hydraulically bound base layers and road sub-bases.

The plants authorized for the recovery of EAF-C slag typically operate on the material crushing, grinding and screening operations to obtain grain size curves requests, deferrization possibly in several phases, and in some cases stabilization/maturation, wetting, washing. It would be possible to use other operations, including innovative ones, which in any case must be provided and described in the individual authorizations. Among these latest treatments, the accelerated carbonation allows for storing CO_2_ in a solid and thermodynamically stable form and allows to significantly improve mechanical properties [62–64] with different performances depending on the carbonation methods [65, 66].

Table 2 shows the main characteristics of the treatment plants directly involved in the present work: the accepted EWC codes for EAF slag, the different areas for storage/treatment of waste and storage of final products and a brief description of the treatment processes performed at each plant.

**Table 2:** Main characteristics of the treatment plants directly involved in the present work

Treatment plant	EAF slag EWC codes	Areas	Facilities and treatments
#1	10.02.01, 10.02.02, 10.09.03	• Storage/processing areas for incoming and intermediate waste;	• Crushing/deferrization/screening/selection plant, to obtain CE 2 + certified unbound aggregates, in different sizes and in accordance with the UNI EN technical standards
		• Storage areas for final products	• Mixing plant for the production of cement and/or bituminous mixes and concretes with predefined strengths
#2	10.02.02, 10.09.03	• Storage/processing areas for incoming and intermediate waste;	• Initial manual selection to remove fractions unsuitable for subsequent treatments
		• Storage areas for final products	• Crushing/deferrization/screening plant (all aggregates are marked CE 2 + , in accordance with the UNI EN technical standards)
#3	10.02.01, 10.02.02, 10.09.03	• Storage/processing areas for different incoming waste (C&D, steel slags, milled asphalt, etc.);	• Crushing/deferrization/screening/selection plant, to obtain CE and CE 2 + certified unbound aggregates in different sizes and in accordance with the UNI EN technical standards;
		• Storage areas for final products	• Bituminous mix production plant;
			• Small plant for the production of concrete with natural and artificial aggregates
#4	10.02.01, 10.02.02, 10.09.03	n.d	• Crushing/screening/particle-size selection/deferrization plant;
			• Plants for mixing aggregates with hydraulic binders (cement mixes), complying with the UNI EN technical standards;
			• Treatments on materials received from third parties and qualified as by-products/EoW: grinding/screening/granulometric selection, in order to obtain aggregates fractions having selected particle size

**Fig. 12:**
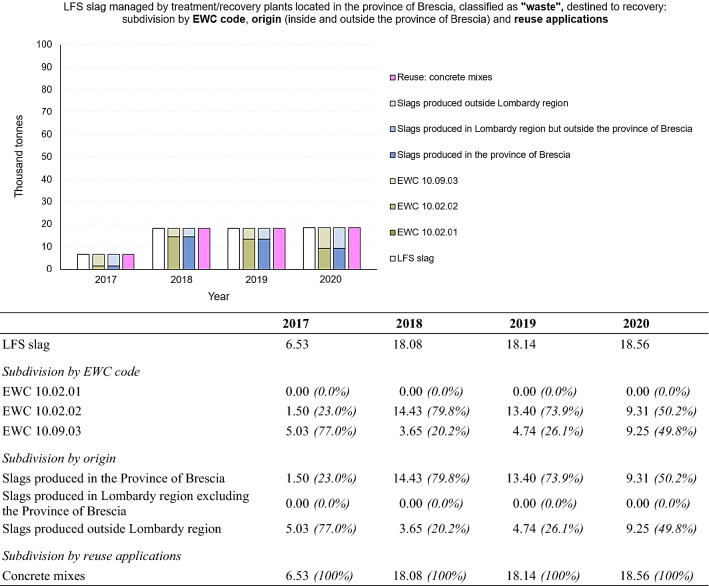
LFS slag managed by treatment/recovery plants located in the Province of Brescia, classified as “waste”, destined to recovery: subdivision by EWC code (10.02.01, 10.02.02, 10.09.03), origin (produced in the province, produced in Lombardy region excluding the Province of Brescia and produced outside Lombardy region) and reuse applications. Period 2017 – 2020. For a better comprehension, the figure is accompanied by a table showing numerical values (in thousand tonnes) and percentages of the total, for each subdivision made

The same data processing and elaborations were carried out for LFS slag (or white slag) from steel refinements in ladle furnaces (Fig. 12). As for EAF slag, the majority of LFS slag are classified with EWC code 10.02.02 (“*unprocessed slags*”), with increasing percentages of slag classified with EWC code 10.09.03 (“*furnace slags*”) from 2017 to 2020 and mostly coming from production within the Province of Brescia.

The small quantities recovered of this type of slag can be justified by their poor physical, chemical and mechanical characteristics (high volumetric instability, tendency to self-pulverize during the cooling process, low hydraulic properties, etc.), which make them unsuitable for the majority of reuses for which EAF slag are suitable [4]. LFS presents hydraulicity that provides it with slightly cementitious properties [67, 68], thus the addition of LF slag is mainly explored when preparing Portland cement mixtures [44, 67, 69]. The potential of LFS in building and construction research has also been studied, mainly to replace cement and lime in varied applications, such as mortars and concrete [69, 70], plasterboard [71], and soil stabilization [72, 73], among others. In any case one of the issues related to the reuse of this slag is its potential expansion.

Because of these poor properties, LFS slag can be reused, if properly treated, as a partial replacement of the binder in civil engineering applications (preferably where the required strength is rather low), soil stabilization, fertilizers or unrestrained construction applications (where the expansion phenomena are less problematic) [4]. From the data obtained and processed, all the LFS slag recovered in the Province of Brescia are reused in concrete mixes.

#### 4.3.2. Recovered Slags Classified as “by-Products”

**Fig. 13:**
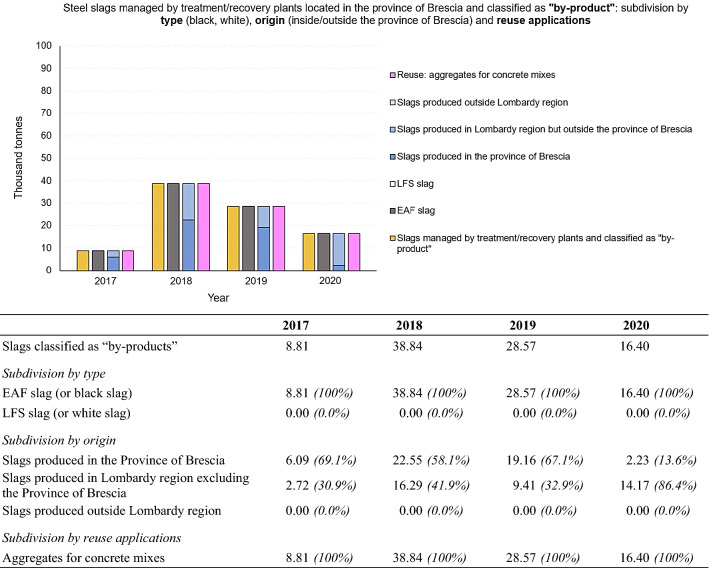
Steel slags managed by treatment/recovery plants located in the Province of Brescia and classified as “by-products”: subdivision by type (EAF/LFS), origin (produced in the province, produced in Lombardy region excluding the Province of Brescia and produced outside Lombardy region) and reuse applications. Period 2017 – 2020. For a better comprehension, the figure is accompanied by a table showing numerical values (in thousand tonnes) and percentages of the total, for each subdivision made

As far as the part of steel slags classified as “by-product” is concerned, Fig. 13 shows their subdivision according to type (EAF/LFS), origin (produced in the Province of Brescia, produced in the Lombardy region (excluding the Province of Brescia) or produced outside the region) and fields of reuse. In contrast to slags classified as “waste”, slags classified as “by product” are mainly treated directly by the producer, most often without the need to involve third parties. After common post-spill treatments (characterization of the chemical composition, cooling, solidification, crushing/grinding, deferrization), an EWC code is assigned to the slags classified as “waste” and producers have to prepare and fill in the necessary declarations (MUD, etc.). “Waste” slags can then be transferred to authorised depot/storage facilities (e.g. treatment/recovery plants for “End of Waste” operations) or disposed in landfills [58]. The “by-products”, after the common post-spill treatments, undergo further crushing/grinding and screening operation, and division into heaps, directly at steel mills. Once registered with ECHA and CE-marked, the by-products can be directly placed on the market, without passing through a treatment/recovery plant [58]. This explains the small quantities shown in Fig. 13, as the treatment/recovery plants are the subject of this analysis.

Of the few quantities of by-products shown in Fig. 13, all are EAF slag, mostly produced in the Province of Brescia and reused as aggregates for the concrete production (after having undergone, in the recovery plant, simple treatments such as grinding, screening and granulometric selection to obtain aggregate fractions with a suitable and selected granulometry).

As mentioned above, not all the companies in the steel sector contacted for the survey participated actively. In addition to the treatment/recovery plants, some steel mills also took part in the questionnaires. Their contributions unfortunately do not represent percentages that would allow a detailed analysis like the one carried out for the treatment/recovery plants. However, the processing of these data also showed that the slags classified as “by-products” are almost exclusively EAF slag and that are reused not only for the production of certified products (e.g. aggregates for concrete production), but also for backfilling, yards, embankments and draining layers for the covering of landfills.

The combined analysis on dta provided by companies in the steel sector.

Analysis of data from one of the four treatment/recovery plants considered revealed small quantities of incoming EAF slag classified as “End of Waste” (about 11 thousand tonnes, Fig. 8). These slag have already undergone appropriate recovery treatments at the steelworks itself or by third parties (depending on the waste treatment and recovery policy adopted by the producer), in order to cease being classified as waste. This small percentage of slag entering the treatment plant and already classified as “End of Waste”, undergoes simple grinding, screening and granulometric selection treatments to obtain inert fractions of a suitable and selected granulometry. After these treatments, they will be marketed as industrial aggregates for concrete mixes, in accordance with the sector technical standards.

## 5. Conclusions

The problem of proper production, management, recovery, disposal and reuse of steel slags is certainly highly topical, especially in areas with high production, such as the Province of Brescia, Italy. Analysis, processing and integration of big data from different sources (regional and provincial databases, sector consortia and questionnaires directly submitted to companies) are therefore essential in order to identify and subdivide the different quantities according to classification, type, origin and final applications. This allows to obtain useful information for the individuation of critical issues and bottlenecks that hinder recovery and reuse and to individuate paths of industrial symbiosis that can be established among local companies.

With specific reference to the analysis conducted in the province of Brescia, results of the big data processing showed the following:

- According to the MUD database, a small percentage the total amount of slags managed as waste in the Province of Brescia (from both internal and external production) was destined for recovery operations in authorized plants (18% in 2017 and 25% in 2018) while the remaining part was destined for disposal in landfills. Even considering the quantities of waste that are classified as by-products, whose data was obtained from the the sector consortia, the disposal percentages remain high and a large amount of steel slag is unfortunately still destined to landfills. In order to reduce this amount, research should focus on use higher percentages of slags for applications for which they are already known to be suitable (road construction, concrete and bituminous mixtures, hydraulically bound base layers, etc.) and, at the same time, studying their possible reuse in innovative fields that are already being tested (e.g. applications of EAF slag in polymer matrices, etc.) or are still being studied (e.g. slag fillers in the field of plasma-applied hard covering, 3D printing, etc.).
- The amount of slag classified as by-product still appear low highlighting critical issues still present in Italy that hinder producers in managing these residues such as the difficulty of demonstrating the condition for by-products relating to the certainty of use (condition a) under the Waste Framework directive (WFD) article 5). As consequence producers prefer divert their residues to dedicated treatment plants. In this context, industrial symbiosis initiatives at regional or national level, supported by a planning actions using MFA, will allow establish industrial symbiosis network among various companies achieving sustainable commercial opportunities that demonstrate the certainty of reuse.
- According to the data obtained from questionnaires, the total amount of steel slags to be managed at the treatment/recovery plants located in the Province of Brescia is almost entirely EAF slag (above 90%) and comes mainly from production within the province (between 80 and 90%). Of the slags classified as “waste” and destined for recovery, almost all of them are EAF slag (more than 90%). There are also small percentages of LFS slag destined for recovery, although their physic-chemical, mineralogical and performance characteristics make their reuse very difficult.
- EAF slag destined for recovery comes almost entirely from production within the province (above 80%) and are mainly reused for hydraulically bound base layers and road foundations (over 70% of the total) and as aggregates in the production of bituminous and cement mixes. On the other hand, the origin of LFS slag destined for recovery is more uneven, with rather significant percentages also from production outside the Lombardy region and are instead reused as partial replacement of the binder in the concrete production;
- The processing of data obtained directly from few steel mills located in the Province of Brescia showed that the slags classified as “by-products” and managed directly in the steelmaking plants are almost exclusively EAF slag. Once placed on the market, they were reused not only for the production of certified products (e.g. aggregate from slags for concrete production), but also for backfilling, yards, embankments and draining layers to cover landfills.
- In addition to slags classified as “waste” and “by-products”, small quantities of EAF slag classified as “End of Waste” have been identified as entering treatment/recovery plants. After simple treatments, they have been reused as industrial aggregates for concrete mixes, in accordance with the sector technical standards.

The present investigation shows as to ensure a complete and proper monitoring of the slags flows (in particular for those classified as “by-products”), it would be advisable to integrate existing systems (e.g. the MUD database) or to set up special registers (e.g. similar to the MUD database).

The proposed methodology developed for the analysis of the steel slags management, combining both information from regional databases and specific survey on all the actors of the recovery chain, allows to overcome the limitations of the regional database, which alone would not allow to obtain disaggregated and detailed information on the type of waste, on the quantities classified as by-products and on the types of destination/reuse. The applied methodology therefore proved to be a useful tool for monitoring flows, the state of implementation of recovery and reuse and industrial symbiosis on regional and provincial scales, and it can be used to plan and implement policies, strategies and Industrial Symbiosis at local, regional or national scales.

## Data Availability

Enquiries about data availability should be directed to the authors.
